# Re: Shedding light on polypragmasy of pain after transurethral prostate surgery procedures: a systematic review and meta-analysis

**DOI:** 10.1007/s00345-021-03663-z

**Published:** 2021-05-22

**Authors:** Jeremy Yuen-Chun Teoh, Marcelo Langer Wroclawski, Steffi Yuen, Bryan Cheng, Riccardo Bertolo, Daniele Castellani, Vineet Gauhar, Thomas Herrmann

**Affiliations:** 1 Department of Surgery, S.H. Ho Urology Centre, The Chinese University of Hong Kong, Shatin, Hong Kong SAR China; 2 Hospital Israelita Albert Einstein, Sao Paulo, Brazil; 3 BP, a Beneficencia Portuguesa de Sao Paulo, Sao Paulo, Brazil; 4 Department of Surgery, United Christian Hospital, Kwun Tong, Hong Kong SAR China; 5 Department of Urology, San Carlo di Nancy Hospital, Rome, Italy; 6 Department of Urology, IRCCS INRCA, Ancona, Italy; 7 Department of Urology, Ng Teng Fong General Hospital, NUHS, Singapore, Singapore; 8 Department of Urology, Spital Thurgau AG (STGAG), Pfaffenholzstraße4, 8501 Frauenfeld, Switzerland; 9 Department of Urology, Hanover Medical School (MHH), Hannover, Germany

Dear Editor,

Persistent dysuria, pelvic pain or prostatodynia after transurethral prostate surgery is a classical example of a well recognised but poorly documented urological condition. Clinical presentation can be variable but the symptomatology is somewhat similar to the chronic pelvic pain syndrome [1, 2]. The pain is usually bothersome and it tends to be persistent and refractory to medications. On the other hand, the etiology and pathophysiology are totally different, and the pain always occurs after transurethral prostate surgery. We believe its characteristics are clear and distinct enough to define it as the post-operative pelvic pain syndrome (PPPS).

Recently, we conducted a five-question survey to investigate the real world practice of managing PPPS after transurethral prostate surgery. The five questions covered the (1) current position of the respondent, (2) choice of treatment modality for patients with PPPS after transurethral prostate surgery, (3) choice of anti-inflammatory medications for patients with PPPS after transurethral prostate surgery, (4) duration of treatment before being determined ineffective, and (5) treatment response based on a scale of 0–10, i.e., Out of ten patients, how many would actually respond to the treatment. The survey was primarily distributed via the #UroSoMe Twitter platform [3]. A tweet about prolonged pain after transurethral prostate surgery was posted together with a link which directed to the Google Form survey platform. The survey was launched on 4th October 2020 and lasted for 6 weeks.

A total of 230 responses were received when the survey was concluded. Among the 230 respondents, 80.0% were urology consultants, 9.6% were urology fellows and 10.4% were urology residents in training. Regarding the choice of treatment modality (Table 1), the majority would offer anti-inflammatory agents (88.7%), followed by alpha-blocker (42.2%), gabapentin/pregabalin (40.4%) and pelvic physiotherapy (39.6%). For the choice of anti-inflammatory agents, the majority would offer oral non-steroidal and anti-inflammatory drugs (NSAIDs) (81.3%), followed by NSAIDs suppository (17.0%), oral corticosteroids (17.0%) and intramuscular corticosteroids (8.3%). About half of the respondents (49.1%) would try the treatment for 4 weeks before they determine it to be ineffective, but 27.4% would allow a prolonged treatment duration of 8–12 weeks. In their experiences, a mean of 5.9 out of ten patients would respond to the treatment being given.

**Table 1:** Choice of treatment for patients with post-operative pelvic pain syndrome

	Number of responses (%)
Choice of treatment modality	
Anti-inflammatory medication	204 (88.7%)
Alpha-blocker	97 (42.2%)
Gabapentin / Pregabalin	93 (40.4%)
Pelvic physiotherapy	91 (39.6%)
Anti-cholinergic medication	66 (28.7%)
Antibiotics	64 (27.8%)
Phenazopyridine	25 (10.9%)
Beta-3 agonist	23 (10.4%)
Opioid	20 (8.7%)
Saw palmetto	15 (6.5%)
Sitz bath	15 (6.5%)
Baclofen	13 (5.7%)
Amitriptyline	7 (3.0%)
Low-intensity ESWT	7 (3.0%)
Serratiopeptidase	4 (1.7%)
Anti-fungals	2 (0.9%)
Tadalafil	1 (0.4%)
Choice of anti-inflammatory medications	
Oral NSAIDs	187 (81.3%)
NSAIDs suppository	39 (17.0%)
Oral corticosteroids	39 (17.0%)
Intramuscular corticosteroids	19 (8.3%)
Corticosteroids suppository	9 (3.9%)
Intravenous corticosteroids	4 (1.7%)

*ESWT* extracorporeal shock wave therapy, *NSAIDs* non-steroidal anti-inflammatory drugs

Based on the above survey results, a recommendation on how to manage patients presenting with persistent dysuria/pelvic pain/prostatodynia after transurethral prostate surgery was developed.

1. Rule out post-operative complications and organic causes such as urinary tract infection and capsular / bladder perforation.
2. Allow at least 8–12 weeks for the patient to recover from surgery before confirming the diagnosis of PPPS.
3. Pelvic physiotherapy can be offered early given the non-invasive nature of the treatment [4, 5].
4. In addition, concomitant use of anti-inflammatory medications can be considered [6, 7]. Oral NSAIDS, NSAIDS suppository and oral corticosteroids are the preferred choice of anti-inflammatory medications.
5. If anti-inflammatory medications fail, other medications such as alpha-blockers and gabapentin / pregabalin can be considered at a later stage in a sequential manner [6, 7].
6. A minimum of 4 weeks and a maximum of 3 months should be allowed for the effects of each medication to take place [8–10].

Although this is just a short survey with five questions, it pinpoints the most important aspects in managing PPPS. The survey results represent the preferred management from urologists worldwide and can be considered an implied consensus regarding the best management of PPPS. In addition to this survey, a systematic review on PPPS after transurethral prostate surgery is currently under preparation. We hope to provide the ‘best evidence’ that we have so far and we believe it will be useful for providing guidance in in managing this condition. Although PPPS is not very common, it is certainly a very bothersome condition that deserves more thorough investigations. The optimal management of PPPS should be considered a priority and high-quality research work is urgently needed in the near future.
